# Hip abductor moment arm - a mathematical analysis for proximal femoral replacement

**DOI:** 10.1186/1749-799X-6-6

**Published:** 2011-01-25

**Authors:** Eric R Henderson, German A Marulanda, David Cheong, H Thomas Temple, G Douglas Letson

**Affiliations:** 1 Department of Orthopaedics and Sports Medicine, 13220 Laurel Drive, University of South Florida, Tampa, Florida, 33612, USA; 2 Sarcoma Division, 12902 Magnolia Drive, H. Lee Moffitt Cancer & Research Institute, Tampa, Florida, 33612, USA; 3 Orthopaedic Oncology Division, Department of Orthopaedic Surgery, University of Miami, Miami, Florida, USA

## Abstract

**Background:**

Patients undergoing proximal femoral replacement for tumor resection often have compromised hip abductor muscles resulting in a Trendelenberg limp and hip instability. Commercially available proximal femoral prostheses offer several designs with varying sites of attachment for the abductor muscles, however, no analyses of these configurations have been performed to determine which design provides the longest moment arm for the hip abductor muscles during normal function.

**Methods:**

This study analyzed hip abductor moment arm through hip adduction and abduction with a trigonometric mathematical model to evaluate the effects of alterations in anatomy and proximal femoral prosthesis design. Prosthesis dimensions were taken from technical schematics that were obtained from the prosthesis manufacturers. Manufacturers who contributed schematics for this investigation were Stryker Orthopaedics and Biomet.

**Results:**

Superior and lateral displacement of the greater trochanter increased the hip abductor mechanical advantage for single-leg stance and adduction and preserved moment arm in the setting of Trendelenberg gait. Hip joint medialization resulted in less variance of the abductor moment arm through coronal motion. The Stryker GMRS endoprosthesis provided the longest moment arm in single-leg stance.

**Conclusions:**

Hip abductor moment arm varies substantially throughout the hip's range of motion in the coronal plane. Selection of a proximal femur endoprosthesis with an abductor muscle insertion that is located superiorly and laterally will optimize hip abductor moment arm in single-leg stance compared to one located inferiorly or medially.

## Background

**Figure 1:**
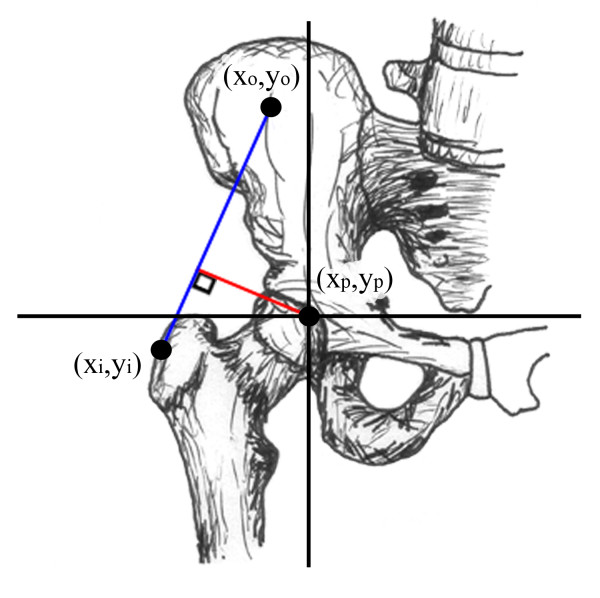
**Coronal view of hip demonstrating hip abductor moment arm, (red line)**. Coronal view of hip demonstrating hip abductor moment arm, defined as the length of a line originating at the joint center (red) which forms a 90˚ angle with the line of action (blue).

Proximal femoral reconstruction is a challenging procedure that is commonly indicated in orthopaedic oncology, complex hip revision surgery, and trauma [1,2]. The replacement of the proximal femur irreversibly affects the normal anatomy and biomechanics of the hip joint. A Trendelenberg gait is the most common reported complication of proximal femoral replacement [1-7]. In addition, falling is a common source of postoperative morbidity and has been linked to postural instability and muscle weakness in the single leg stance [8,9]. Lower extremity strength and standing balance have also been shown to be predictive of disability [10]. Johnston et al reported that there are three hip factors that determine the occurrence of a limp [11]. The first factor is the moment (torque) that a given muscle must generate. The second factor is the length of the moment arm of that muscle and the third is the strength of the given muscle. The moment arm of a given muscle (effective lever arm) is the length of a straight line originating at the joint center (femoral head), and terminating at a point 90° to the muscle's line of action (Figure 1).

The greater trochanter in the normal femur serves as the insertion point for the hip abductor muscles, gluteus medius and gluteus minimus. Normal function of these muscles is required for single-leg stance and ambulation [11-15]. Altering the site of the insertion of the abductor muscles, as is seen with proximal femoral replacement, significantly affects hip biomechanics [11-15].

Muscle moment arm is usually discussed as a static quantity. Motion at the hip joint, however, requires the femur to move relative to the pelvis, resulting in alterations in abductor moment arm with gait [13]. Evaluating hip abductor moment arm as the hip travels through its coronal range of motion has not been performed previously. The purpose of this study was to analyze the abductor moment arm characteristics through hip adduction and abduction. In addition, the authors will provide an objective evaluation of the clinical and mechanical advantages afforded by specific alterations in patient anatomy and commercially-available proximal femoral endoprostheses.

## Methods

A mathematical model of abductor moment arm, defined by anatomical coordinates of the origins and insertions of the gluteus medius and minimus and the femoral head center, was derived using anatomical measurements published previously (Figure 1) [11,13,16]. The straight line method of approximating the path of muscle pull was used for this study since the broad attachments of the gluteus medius and gluteus minimus do not facilitate definition of the transverse sections required for the centroid line model [11,16,17].

Derivation of the mathematical model began with the equation for moment arm (Equation 1, see Appendix). Moment arm was calculated by defining the lever arm (r) in terms of the femoral head and abductor muscle insertions (Equation 2), and defining the angle of pull (θ) in terms of the femoral head, muscle origins, and insertions (Equation 3). The moment arm could therefore be calculated and plotted for all values of a muscle's origin, insertion, and joint center (Equation 4).

The moment arm of the normal femur was calculated and plotted from 30° of adduction through 45° of abduction using mathematics software (Maplesoft, Ontario, Canada), which was then employed for all further analyses [16]. Modifications of the greater trochanter that were analyzed and plotted included several modes of displacement: two centimeters (cm) of lateral, medial, superior, inferior, or supero-lateral displacement. Modifications of the moment arm equation were required for this analysis (Equations 5 and 6).

**Figure 2:**
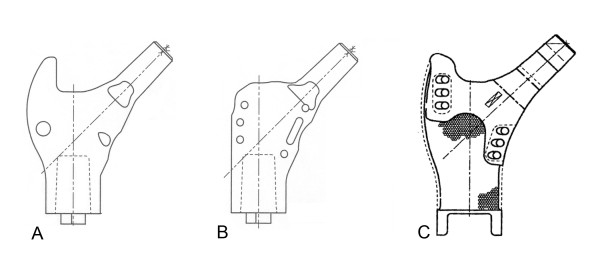
**Proximal femoral prostheses**. Schematics of commercially available proximal femoral prostheses: A. Biomet Letson; B. Biomet Finn; C. Stryker GMRS.

The second analysis compared abductor moment arm through 30° of adduction and 45° of abduction for three commercially-available proximal femur prostheses and the normal femur. These prostheses were the Biomet 7 cm Letson Proximal Component, Biomet 7 cm Finn Proximal Component, (Biomet Orthopedics, Warsaw, Indiana, USA) and the Stryker Global Modular Replacement System with greater trochanter (GMRS, Stryker Orthopedics, Mahwah, New Jersey, USA). The design data for the prostheses were obtained from schematics provided by the manufacturers (Figure 2).

**Figure 3:**
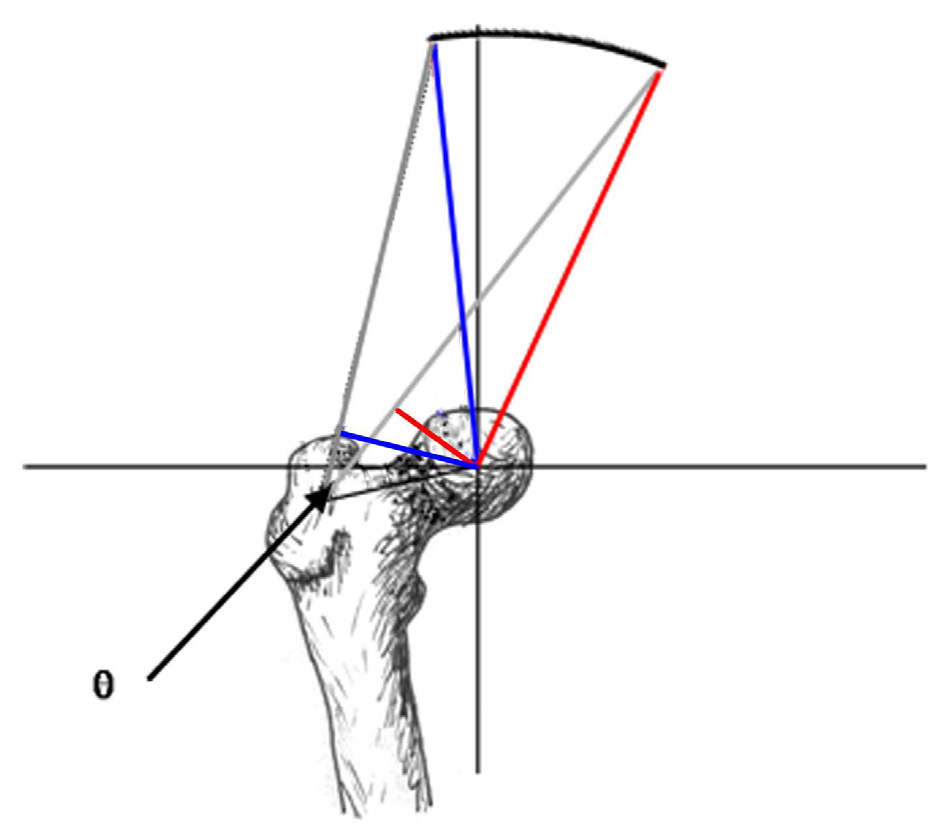
**Simulation of pelvic tilt**. Graphic depiction of the normal relationship of the abductor muscle origin in relation to the femoral head (blue lines) and the position of hip abductor muscle origin with 30° pelvic tilt (red lines).

**Figure 4:**
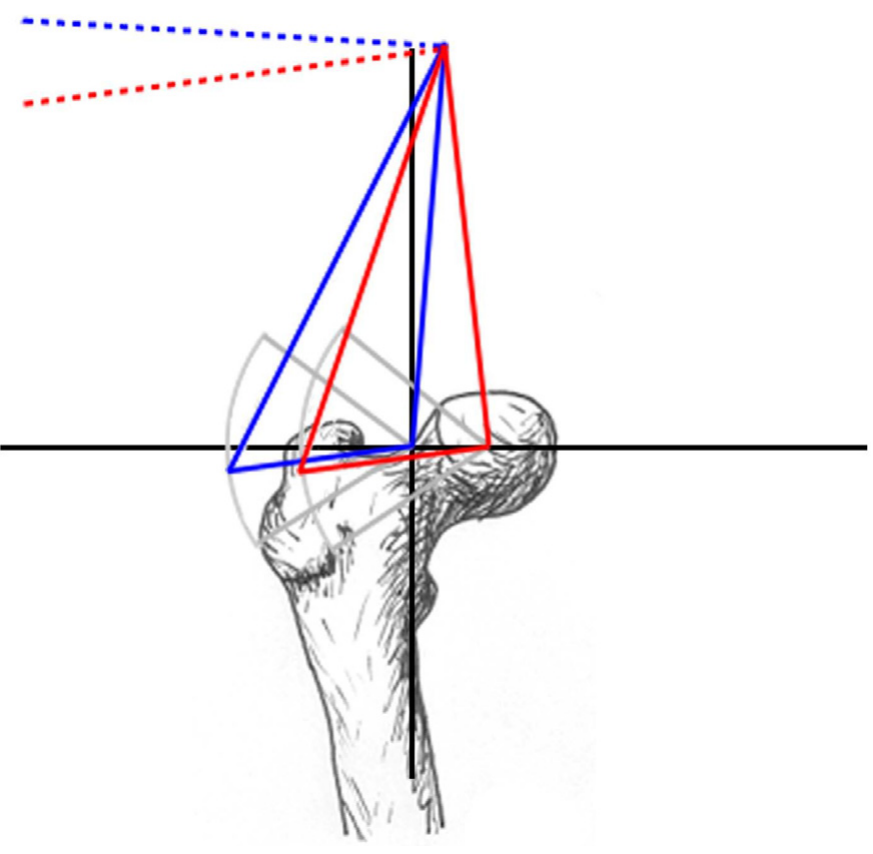
**Proximal femoral prostheses**. Graphic depiction of the normal relationship of the abductor muscle insertion in relation to the femoral head (blue lines) and the position of hip abductor muscle insertion with the femoral head after medial displacement (red lines). The dotted lines represent the trajectory along which the abductor muscle moment arm would be maximal.

Abductor moment arm was also analyzed in the setting of abductor muscle weakness and a Trendelenberg gait, simulated as tilting of the pelvis away from the affected joint (Figure 3). An additional modification of the moment arm equation was required for this analysis (Equations 7 and 8). The final analysis examined the effect of medialization of the proximal femur on abductor moment arm. This study required a change in coordinates of the femoral head and the greater trochanter (Figure 4).

## Results

**Figure 5:**
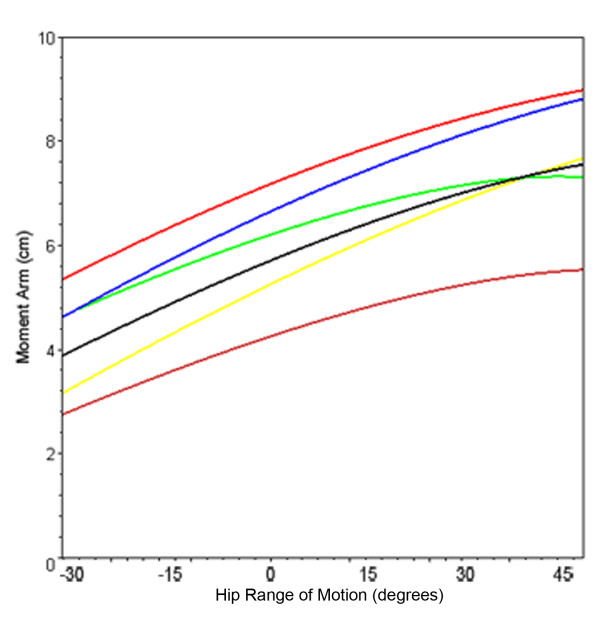
**Hip abductor moment arm plot for normal femur**. Line plot showing mean hip abductor moment arm through coronal plane motion for the normal femur (black) and femurs with greater trochanter displacements 2 cm medial (orange), 2 cm inferior (yellow), 2 cm (superior) green, 2 cm lateral (blue), 2 cm superior and 2 cm lateral (red).

The abductor moment arm of the normal femur was 5.6 centimeters in neutral standing position (Table 1). Moment arm was greatest with superolateral displacement of the greater trochanter through the entire range of hip motion in the coronal plane (Figure 5). Equal moment arm lengths occurred with isolated superior displacement or lateral displacement at maximum adduction. As the hip ranged into abduction, the laterally displaced greater trochanter had a considerably larger moment arm (8.8 centimeters), exceeding the superiorly displaced greater trochanter by 21%. Inferior displacement of the greater trochanter substantially decreased abductor mechanical advantage in adduction, but as the hip ranged into abduction it exceeded the moment arm of both the normal and the superiorly displaced greater trochanters. Medial displacement of the greater trochanter resulted in the smallest moment arm for the entire range of motion.

**Table 1:** Abductor Moment Arm for Commerical Prostheses and Native Femur

	Muscle Division	Position	Abductor Moment Arm (cm)			
			
			Biomet - Letson	Biomet - Finn	Stryker - GMRS	Native Femur
		**30° Adduction**	**4.2**	**3.6**	**4.5**	**4.5**
	**Anterior**	Neutral	4.9	4.8	5.4	6.0
		45° Abduction	3.5	5.2	4.4	7.4
	
		30° Adduction	3.6	2.9	3.8	3.7
**Gluteus Medius**	Middle	Neutral	4.7	4.3	5.1	5.4
		45° Abduction	4.6	5.4	5.4	7.2
	
		30° Adduction	2.8	2.0	2.9	2.6
	Posterior	Neutral	4.0	3.5	4.2	4.2
		45° Abduction	5.0	5.0	5.5	6.2

		30° Adduction	4.6	4.1	5.0	5.5
	Anterior	Neutral	5.0	5.2	5.6	7.1
		45° Abduction	2.5	4.5	3.0	7.3
	
		30° Adduction	3.8	3.0	4.0	4.0
Gluteus Minimus	Middle	Neutral	4.8	4.5	5.3	6.1
		45° Abduction	4.3	5.4	5.0	7.8
	
		30° Adduction	2.7	1.8	2.8	2.4
	Posterior	Neutral	4.0	3.4	4.3	4.5
		45° Abduction	5.0	5.2	5.6	6.9

		30° Adduction	3.6	2.9	3.8	3.8
Mean	All Divisions	Neutral	4.6	4.3	5.0	5.6
		45° Abduction	4.2	5.1	4.8	7.1

**Figure 6:**
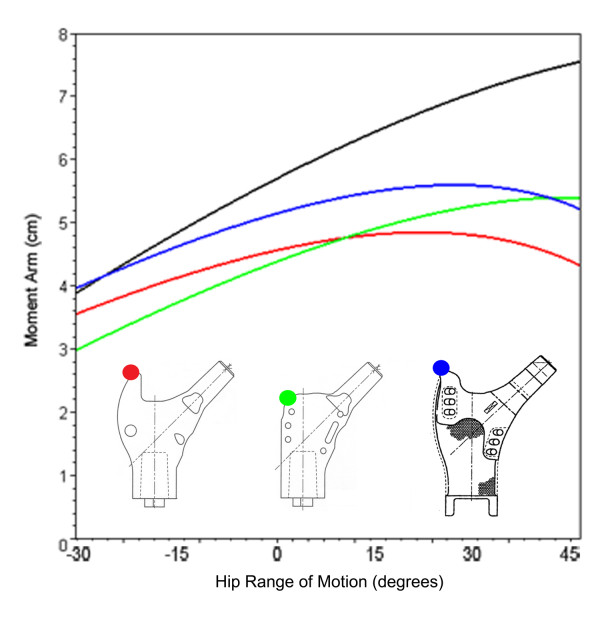
**Hip abductor moment arm plot for proximal femur prostheses**. Line plot showing mean hip abductor moment arm through coronal plane motion for the normal femur (black), Biomet Finn prosthesis (green), Biomet Letson prosthesis (red), and Stryker GMRS prosthesis (blue).

Similar to the native femur, the inferior placement of the abductor muscle insertion site of the Biomet Finn proximal femur endoprosthesis yielded a decreased abductor moment arm (4.3 cm) in neutral hip position (Table 1). This value was lowest in adduction (2.9 cm) and increased as the hip ranged into abduction (5.1 cm). The Biomet Finn exceeded the moment arm of the Biomet Letson at 10° of abduction and exceeded the Stryker GMRS at 40° of abduction (Figure 6). The Stryker GMRS prosthesis had the largest moment arm in neutral position (5.0 cm), 16% greater than the Finn and 9% greater than the Letson (4.6 cm).

The GMRS and Biomet Letson prostheses, which have prominences for abductor muscle insertion that are superolateral to the abductor attachment site of the Finn, had increased moment arm lengths when abducted from neutral position, however, their values peaked at 27° and 22° of abduction, respectively, and then decreased. Moment arm length for the Finn model increased throughout abduction (Figure 6).

**Figure 7:**
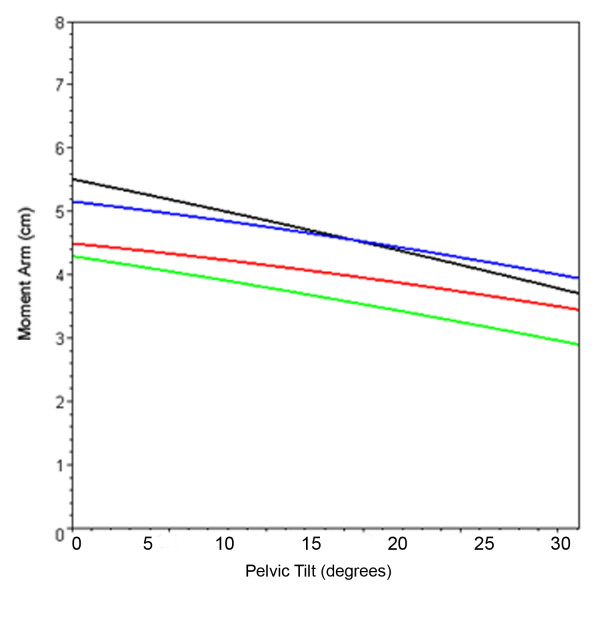
**Effect on hip abductor moment arm with pelvic tilt**. Line plot showing mean abductor moment arm of the hip joint in neutral position through 30˚ of pelvic tilt for the normal femur (black), Biomet Finn prosthesis (green), Biomet Letson prosthesis (red), and Stryker GMRS prosthesis (blue).

Increasing degrees of pelvic tilt, as seen with Trendelenberg gait, caused a mean decrease in abductor moment arm for all hip configurations, with the most substantial differences seen with inferiorly-placed abductor insertions (Figure 7). Abductor moment arm values through 30° of pelvic tilt were equivalent to values through 30° of adduction as these motions result in the same relative geometric changes between femur and pelvis (Table 2).

**Table 2:** Abductor Moment Arm in Neutral Stance with 30° Pelvic Tilt

	Muscle Division	Abductor Moment Arm (cm)			
		
		Biomet - Letson	Biomet - Finn	Stryker - GMRS	Native Femur
	Anterior	4.2	3.6	4.5	4.5
	
Gluteus Medius	Middle	3.6	2.9	3.8	3.7
	
	Posterior	2.8	2.0	2.9	2.6

	Anterior	4.6	4.1	5.0	5.5
	
Gluteus Minimus	Middle	3.8	3.0	4.0	4.0
	
	Posterior	2.7	1.8	2.8	2.4

Mean		3.6	2.9	3.8	3.8

Change from 0° Tilt		-22%	-33%	-24%	-32%

**Figure 8:**
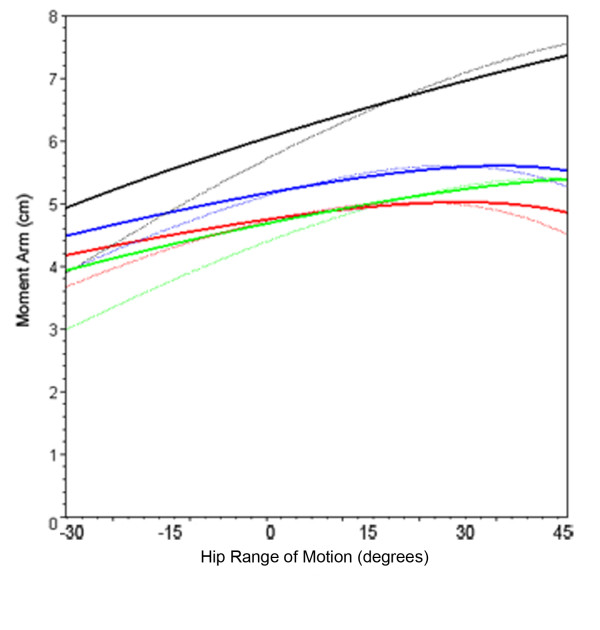
**Hip abductor moment arm with femoral head medialization**. Line plot showing mean hip abductor moment arm with medialized femoral head through coronal plane motion for the normal femur (black), Biomet Finn prosthesis (green), Biomet Letson prosthesis (red), and Stryker GMRS prosthesis (blue) - plots of configurations without medialization shown in dashed lines.

Medialization of the femoral head caused a mean increase in abductor moment arm for the adducted hip of 23% for all hip configurations (Table 3); a mean increase of 30% was seen with the Finn and the native femur. Medialization also resulted in a 34% decrease in moment arm variance, creating a plateau effect over the range of coronal plane motion as the simulated femur moved from 30° of adduction to 45° of abduction (Figure 8).

**Table 3:** Abductor Moment Arm with Medialization of Femoral Head

	Muscle Division	Position	Abductor Moment Arm (cm)			
			
			Biomet - Letson	Biomet - Finn	Stryker - GMRS	Native Femur
		30° Adduction	4.6	4.5	4.9	5.5
	Anterior	Neutral	5.0	5.0	5.4	6.3
		45° Abduction	4.1	5.4	5.0	7.3
	
		30° Adduction	4.1	3.9	4.4	4.7
Gluteus Medius	Middle	Neutral	4.7	4.6	5.1	5.8
		45° Abduction	4.9	5.4	5.6	7.0
	
		30° Adduction	3.4	3.1	3.6	3.7
	Posterior	Neutral	4.0	3.9	4.3	4.6
		45° Abduction	4.9	4.8	5.2	5.7

		30° Adduction	4.8	4.8	5.3	6.3
	Anterior	Neutral	5.0	5.3	5.6	7.3
		45° Abduction	3.3	4.8	3.8	7.5
	
		30° Adduction	4.2	3.9	4.6	5.1
Gluteus Minimus	Middle	Neutral	4.8	4.8	5.3	6.4
		45° Abduction	4.8	3.4	5.4	7.7
	
		30° Adduction	3.3	2.9	3.5	3.6
	Posterior	Neutral	4.1	3.8	4.4	4.9
		45° Abduction	5.0	5.0	5.5	6.5

		30° Adduction	4.1	3.9	4.4	4.8
Mean	All Divisions	Neutral	4.6	4.6	5.0	5.9
		45° Abduction	4.5	4.8	5.1	7.0

## Discussion

The purpose of this investigation was to characterize hip abductor muscle moment arm through coronal plane motion in the setting of normal anatomy, modified anatomy, and with the use of proximal femur endoprostheses. The authors also sought to analyze abductor muscle moment arm in the setting of abductor weakness.

**Figure 9:**
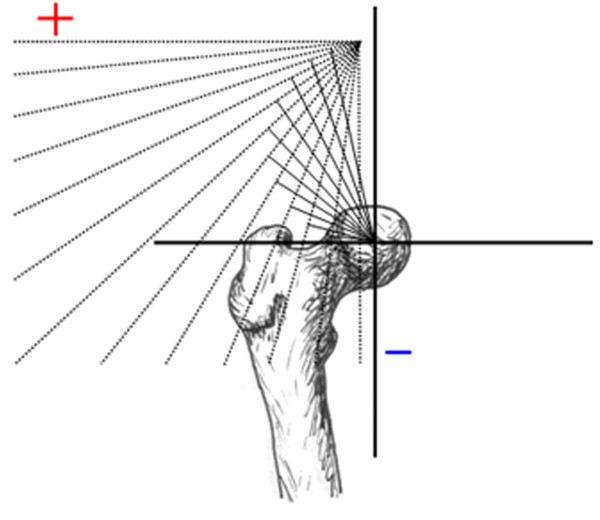
**Coronal view demonstrating potential lines of abductor pull and resultant moment arm lengths**. Coronal view of hip showing potential lines of abductor muscle pull (dotted lines) radiating from a point approximating the middle division of gluteus medius and the corresponding abductor moment arm lengths (solid lines). Movement of the abductors' insertion in the direction of the red positive sign (lateral or superior) would result in lengthening of the abductor moment arm. Movement of the abductors' insertion in the direction of the blue negative sign (medial or inferior) would result in shortening of the abductor moment arm.

Results of the current study demonstrate that hip abductor moment arm is substantially affected by changes in abductor insertion location and coronal plane motion. Because a muscle's moment arm is the length of a line drawn perpendicular to the line of action and intersecting the center of rotation (Figure 1), all muscle insertions along a given line of action will have identical moment arm values, as demonstrated in Figure 9. Superior and lateral displacement of the greater trochanter will move the abductor muscle insertion in a direction that increases moment arm and inferior or medial displacement will move the insertion in a direction that decreases moment arm. Because a line of action must be crossed in order to change the moment arm, combinations of inferior/lateral displacement and superior/medial displacement would produce little or no change in moment arm, as depicted in Figure 9.

**Figure 10:**
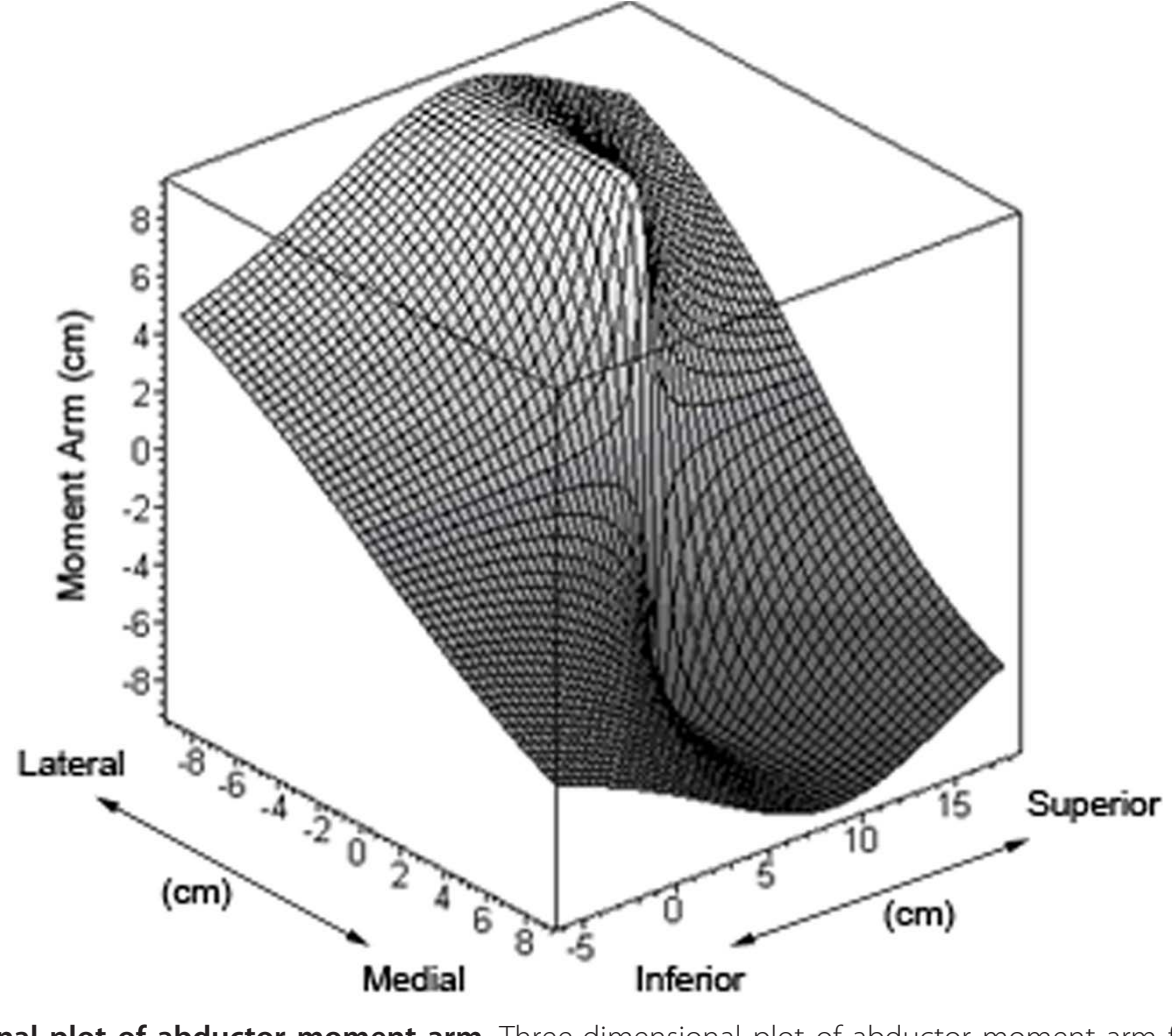
**Three-dimensional plot of abductor moment arm**. Three-dimensional plot of abductor moment arm for a right hip where the x-y projection represents the coronal plane, the hip center is located at (0,0,0), and the z axis represents abductor moment arm. Abductor moment arm achieves a maximum value lateral to the abductor origin and does not increase if the insertion is moved further lateral. Negative moment arm values are possible medially where abductor muscle firing would result in adduction.

A corollary to be drawn from Figure 9 is that the maximum potential hip abductor moment arm can never exceed the distance between the muscle origin and the femoral head. Abductor moment arm attains its maximum value lateral to the origins of the hip abductor muscles and does not increase after this point (Figure 10). The 'negative' values of moment arm noted in Figure 10 indicate the point at which the muscle insertion has moved medial to the femoral head, in which case abductor contraction would result in femur adduction as the muscle would cross the joint on the opposing side, thus resulting in 'negative' abduction.

The curves generated by the different abductor muscle insertion sites (Figures 5 and 6) demonstrate the consequences of greater trochanter manipulation. Inferior displacement of the greater trochanter from its position on the normal femur will result in a decrease in moment arm in neutral position. As the leg abducts the moment arm of the inferiorly displaced greater trochanter increases and will eventually exceed that of the abducted, unmodified greater trochanter. This occurs because the axis of the femoral head and inferiorly displaced greater trochanter (Figure 1) intersects the line of muscle pull at an angle less than 90°. As the leg abducts the angle is increased, thereby increasing the moment arm. Conversely, a superiorly-placed abductor insertion will have a moment arm that exceeds that of the unmodified greater trochanter in neutral position. However, the moment arm of the superiorly-displaced greater trochanter will decrease as the leg abducts (since its angle θ is already at or past 90° when the femur is in neutral position). Calculations from the present study show that in neutral position the Stryker GMRS prosthesis provides a moment arm that is 16% greater than the Biomet Finn, indicating that the abductor force requirement to produce a given torque is 16% less with the GMRS model. A displacement of the greater trochanter that creates a longer moment arm with standing and ambulation is associated with an increase in the available resultant hip muscle force and an accompanying decrease in joint contact force and required resultant force of the hip abductors [12,14]; both of which are associated with a favorable postoperative functional outcome [11,18-20] and a decreased incidence of hip prosthesis failure [21-23].

The present analysis of moment arm in the setting of a Trendelenberg gait demonstrates that superior and lateral placement of the greater trochanter provide greater mechanical advantage through 30° of pelvic tilt. As the pelvis leans away from the affected leg the angle formed by the axes of the femoral neck and muscle fibers becomes more acute and would eventually reach 0°, causing a total loss of abductor effect (Figure 3). An inferiorly-located abductor insertion compromises the abductor muscles prior to rotation of the hip. Given that Trendelenberg gait is a common complication of proximal femoral replacement [1-7], the authors recommend the use of a proximal component with a lateral and/or superior abductor attachment site.

**Figure 11:**
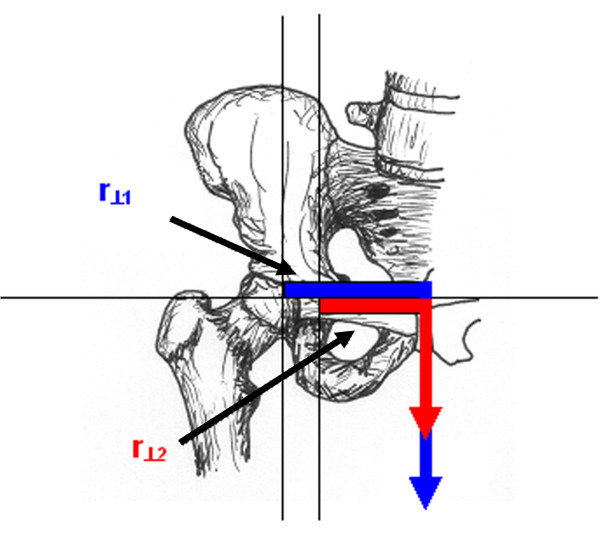
**Resultant hip moment**. Graphic depiction of the normal hip resultant moment (blue line) and the hip resultant moment when the femoral head has been medialized (red line).

Medialization of the femoral head had two effects on moment arm. First, it decreased the distance between joint center and muscle origin thereby lowering the maximum potential moment arm of the abductor unit. Second, the trajectory of the line defining the maximum moment arm was lowered, reducing moment arm variance. Johnston et al. analyzed the hip resultant moment, the abductor muscle force, the hip joint contact force, and the prosthetic neck-stem bending moment in the setting of greater trochanter and hip center manipulation [11]. The authors reported that the movement of the hip center had the greatest effect on all four quantities. All quantities were reduced with medial and inferior placement of the joint center, a favorable result, and were increased with superior and lateral placement of the joint center, which is an unfavorable result. Lateral displacement of the abductor muscle insertion resulted in smaller reductions in all quantities except the joint resultant moment, which remained unchanged. The effect of medial displacement of the hip center on the joint contact force and the resultant hip moment far outweighed those of lateralizing the greater trochanter. This finding seems to contradict the modest advantage in moment arm afforded by movement of the hip center. However, the primary benefit of medial displacement of the hip center has a minimal correlation with increasing the moment arm of the abductor muscles. Instead, this significant advantage is due to the consequent decrease in moment arm and resultant moment of the body itself (Figure 11). This reduces the lever arm and the fraction of bodyweight that contributes to the resultant moment, thereby reducing both components of torque. It is this moment that the abductors must balance in order to maintain a one-legged stance. The authors of the current investigation believe that optimizing functional outcomes in patients undergoing proximal femoral replacement is best achieved with a combination of joint center medialization and selection of a prosthesis that provides the maximum moment arm in single-leg stance.

The authors recognize weaknesses in the current study. The present model is based on previously published coordinates that represent a normal hip. Anatomical variation between patients will cause moment arm values with and without surgical manipulation to vary from our results. Furthermore, our model is a two-dimensional representation of a three-dimensional construct. Preliminary calculations using a three-dimensional model, however, showed no substantial difference from the two-dimensional model findings with a maximum change of 3.4% from the two-dimensional calculations. Other investigations of the hip abductor muscles have confined their analyses to the frontal plane citing similar results [15,24]. Although it is an accepted alternative to abductor-to-prosthesis repair, we did not attempt to simulate soft-tissue attachment of the abductor unit due to the myriad of confounding factors when simulating a viscoelastic medium. The numerical data presented here should not be assumed to be absolute. Instead we wish the surgeon to place emphasis on the concepts and consequences of manipulating native hip joint geometry and how this may be tailored to benefit patients whose compensatory mechanisms or procedure-specific functional prognosis are limited.

## Conclusions

Hip abductor moment arm varies substantially throughout the hip's range of motion in the coronal plane. Lateral and superior movement of the hip abductor muscle insertion will increase moment arm and medial and inferior will decrease their moment arm for single-leg stance. Selection of an endoprosthesis that optimizes hip abductor moment arm will reduce the forces required of the abductor muscles to maintain gait. Reducing the abductor forces required for single-leg stance may help preserve normal ambulation in patients receiving proximal femoral replacement.

## Competing interests

The authors wish to disclose that GDL and HTT are design consultants for Stryker Orthopaedics. The authors also wish to disclose that DC is a design consultant for Salient Surgical Technologies.

## Authors' contributions

ERH derived the mathematical model, conducted the literature search, collected the data, and participated in the writing of the manuscript. GAM and DC provided editorial input and aided in the writing of the manuscript. HTT and GDL oversaw the design of the investigation, construction of the mathematical model, aided with data analysis, and aided with manuscript writing. All authors read and approved the final manuscript.

## Authors' information

ERH - Fifth year orthopaedic surgery resident, University of South Florida, Tampa, USA

GAM - Fourth year orthopaedic surgery resident, University of South Florida, Tampa, USA

DC - Orthopaedic oncologist, Moffitt Cancer Center & Research Institute, Tampa, USA

HTT - Vice Chairman and Orthopaedic oncologist, University of Miami, Miami, USA

GDL - Division Chief and Orthopaedic oncologist, Moffitt Cancer Center & Research Institute, Tampa, USA

## Appendix

(1) $${\text{r}}_{⊥}=\text{r}*\text{sin}\theta$$

Where r is the lever arm length defind as the femoral head-to-abductor insertion point distance in the case of the hip, and θ is equal to the angle between r and the line of muscle pull.

(2) $$\text{r}={\left[{\left({\text{x}}_{\text{p}}-{\text{x}}_{\text{i}}\right)}^{\text{2}}+{\left({\text{y}}_{\text{p}}-{\text{y}}_{\text{i}}\right)}^{\text{2}}\right]}^{\text{0.5}}$$

(3) $$\theta =[\text{arctan}[({\text{y}}_{\text{o}}-{\text{y}}_{\text{i}})/({\text{x}}_{\text{o}}-{\text{x}}_{\text{i}})]-\text{arctan}[({\text{y}}_{\text{p}}-{\text{y}}_{\text{i}})/({\text{x}}_{\text{p}}-{\text{x}}_{\text{i}})]]$$

(4) $${\text{r}}_{⊥}={\left[{\left[{\left({\text{x}}_{\text{p}}-{\text{x}}_{\text{i}}\right)}^{\text{2}}+{\left({\text{y}}_{\text{p}}-{\text{y}}_{\text{i}}\right)}^{\text{2}}\right]}^{\text{0.5}}\right]}^{*}\text{sin}[\text{arctan}[({\text{y}}_{\text{o}}-{\text{y}}_{\text{i}})/({\text{x}}_{\text{o}}-{\text{x}}_{\text{i}})]-\text{arctan}[({\text{y}}_{\text{p}}-{\text{y}}_{\text{i}})/({\text{x}}_{\text{p}}-{\text{x}}_{\text{i}})]]$$

(5) $${\text{x}}_{\text{i}}={\text{r}}^{*}\text{cos}(\text{α}-\beta )$$

(6) $${\text{y}}_{\text{i}}={\text{r}}^{*}\text{sin}(\text{α}-\beta )$$

Where (r * cos α) and (r * sin α) are polar coordinate equivalents for x_i _and y_i_, respectively, and β is the angle of abduction. These substitutions were made for equation 5 and a plot was generated of r_⊥ _as a function of β.

(7) $${\text{x}}_{\text{oi}}={\left[{\left({\text{x}}_{\text{o}}-{\text{x}}_{\text{p}}\right)}^{\text{2}}+{\left({\text{y}}_{\text{o}}-{\text{y}}_{\text{p}}\right)}^{\text{2}}\right]}^{\text{0.5}}*\text{cos}(\chi -\delta )$$

(8) $${\text{y}}_{\text{oi}}={\left[{\left({\text{x}}_{\text{o}}-{\text{x}}_{\text{p}}\right)}^{\text{2}}+{\left({\text{y}}_{\text{o}}-{\text{y}}_{\text{p}}\right)}^{\text{2}}\right]}^{\text{0.5}}*\text{sin}(\chi -\delta )$$

Where χ is the polar coordinate angle for the muscle origin with 0° pelvic tilt and δ is the angle of pelvic tilt. Again, these substitutions were made for equation 5 and a plot was generated of r_⊥ _as a function of δ, whose values ranged from zero ° to 30°.
